# Colonic perforation due to inadvertent intraperitoneal LVAD driveline placement

**DOI:** 10.1186/s13019-020-01240-w

**Published:** 2020-07-28

**Authors:** Ilya Shnaydman, Mohamed O. Abdelhamid, Joyce Kaufman, Howard Lieberman, Gabriel Ruiz

**Affiliations:** Department of Surgery, Ryder Trauma Center, 1800 NW 10th Ave, Miami, FL 33136 USA

**Keywords:** LVAD, Colonic injury, Acute care surgery, Sepsis, Driveline relocation

## Abstract

**Background:**

Left ventricular assist devices (LVAD) are placed for patients with advanced heart failure or cardiogenic shock as destination therapy or as a bridge to cardiac transplantation. Significant complications associated with LVAD placement include bleeding, infection, pump thrombosis, right heart failure, device malfunction and stroke. The case below illustrates inadvertent intraperitoneal driveline placement causing colonic perforation and the subsequent management.

**Case presentation:**

A 54 year old male with a history of Wolff-Parkinson-White syndrome resulting in multiple readmissions for heart failure, ultimately required placement of a left ventricular assist device (LVAD). Several weeks later, he was found to have stool draining from the driveline site. The patient was taken to the operating room for limited exploration by the Cardiothoracic Surgery team and a bowel injury was identified and repaired. Three days after this repair, stool was once again leaking from the driveline site, requiring re-exploration by the Acute Care Surgery team. Intraoperatively, the prior repair was found to be leaking and multiple intra-abdominal abscesses were discovered. The transverse colon was resected and left in discontinuity. On a planned second look operation, the LVAD driveline was relocated to be extra-peritoneal and a colostomy was formed.

**Discussion and conclusion:**

This case demonstrates the importance of early recognition and involvement of an Acute Care Surgeon in the management of this complex problem. Appropriate treatment involves a complete exploration, source control, driveline relocation and possible fecal diversion. Although the incidence of this complication is low, it must be considered in the differential in a septic LVAD patient.

## Background

Despite advances in pharmacotherapy and electrophysiology, heart failure remains a significant cause of morbidity and mortality. Patients who progress to end stage heart failure may be candidates for LVAD as either destination therapy or as a bridge to heart transplantation. LVADs have been shown to have 1 year survival rates as high as 80% [1]. Due to national heart transplant volumes remaining stagnant, there will be a higher number of LVAD implantations. Although LVAD technologies and surgical techniques continue to improve, there are significant complications associated with LVAD placement including bleeding, infection, pump thrombosis, right heart failure, device malfunction and stroke [2]. There are rare reports of intra-abdominal complications of LVAD placement including bowel obstruction, perforation, fistula formation and hernia occurrence [3, 4]. The case below illustrates intraperitoneal driveline placement causing colonic perforation and the subsequent management.

## Case presentation

A 54 year old male with a history of Wolff-Parkinson-White syndrome resulting in cardiac arrest in his 20s, subsequently developed non-ischemic cardiomyopathy (New York Heart Association class IV with an ejection fraction of 15%.) He was admitted for heart failure and had a complicated cardiac care unit course with removal of AICD/Pacemaker due to endocarditis, and anticoagulation with Coumadin for right atrial thrombus/atrial fibrillation. He was discharged, but required multiple readmissions for heart failure, ultimately requiring venoarterial extracorporeal membrane oxygenation through the right femoral artery and left femoral vein as eCPR in January, 2020 and would remain on ECMO for 5 weeks. He underwent LVAD placement using left ventricle and ascending aorta cannulation sites 16 days after ECMO cannulation and subsequent tracheostomy in February for persistent respiratory failure.

**Fig. 1:**
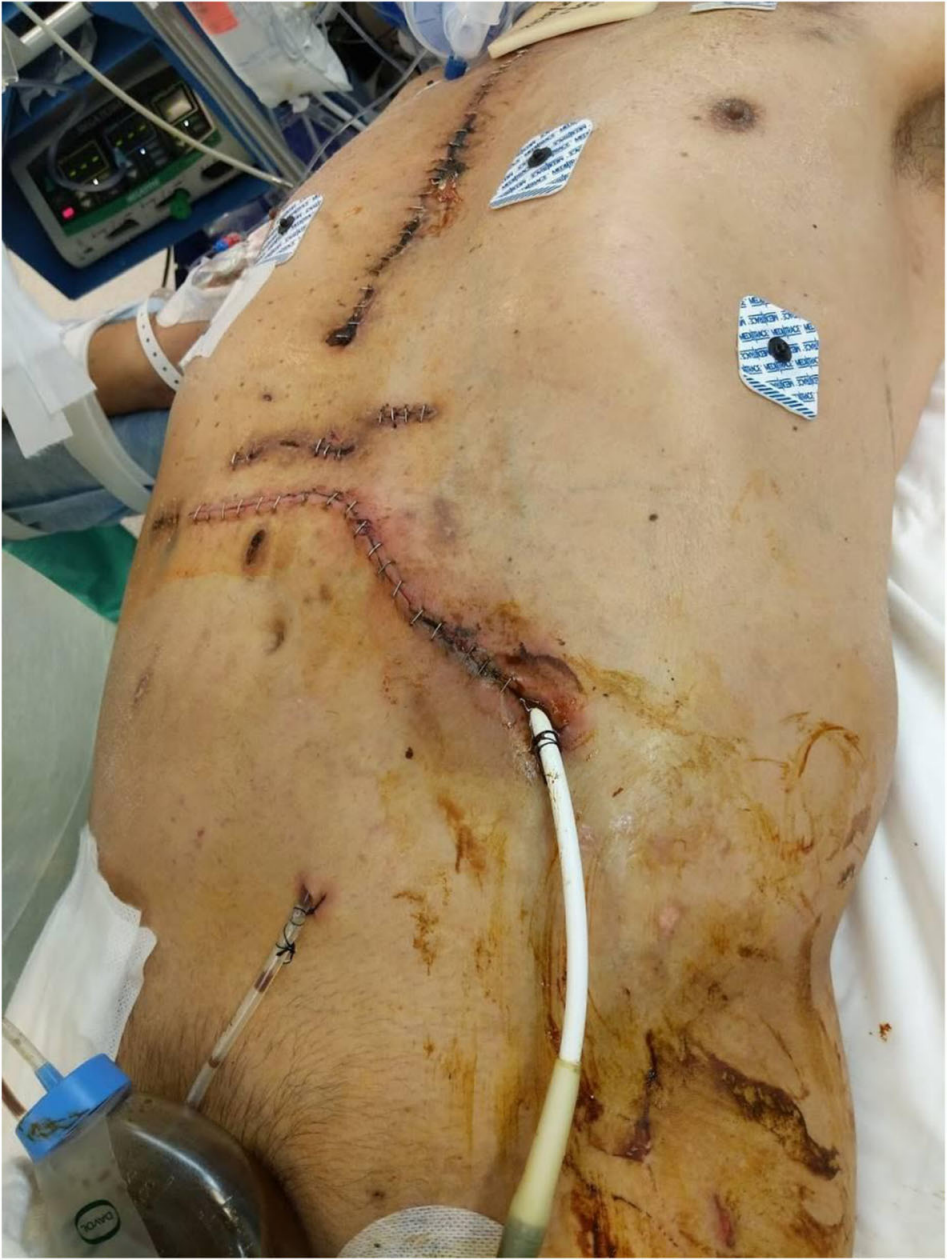
Preoperative photo prior to Acute Care Surgery laparotomy, 3 days after limited exploration by cardiothoracic team

**Fig. 2:**
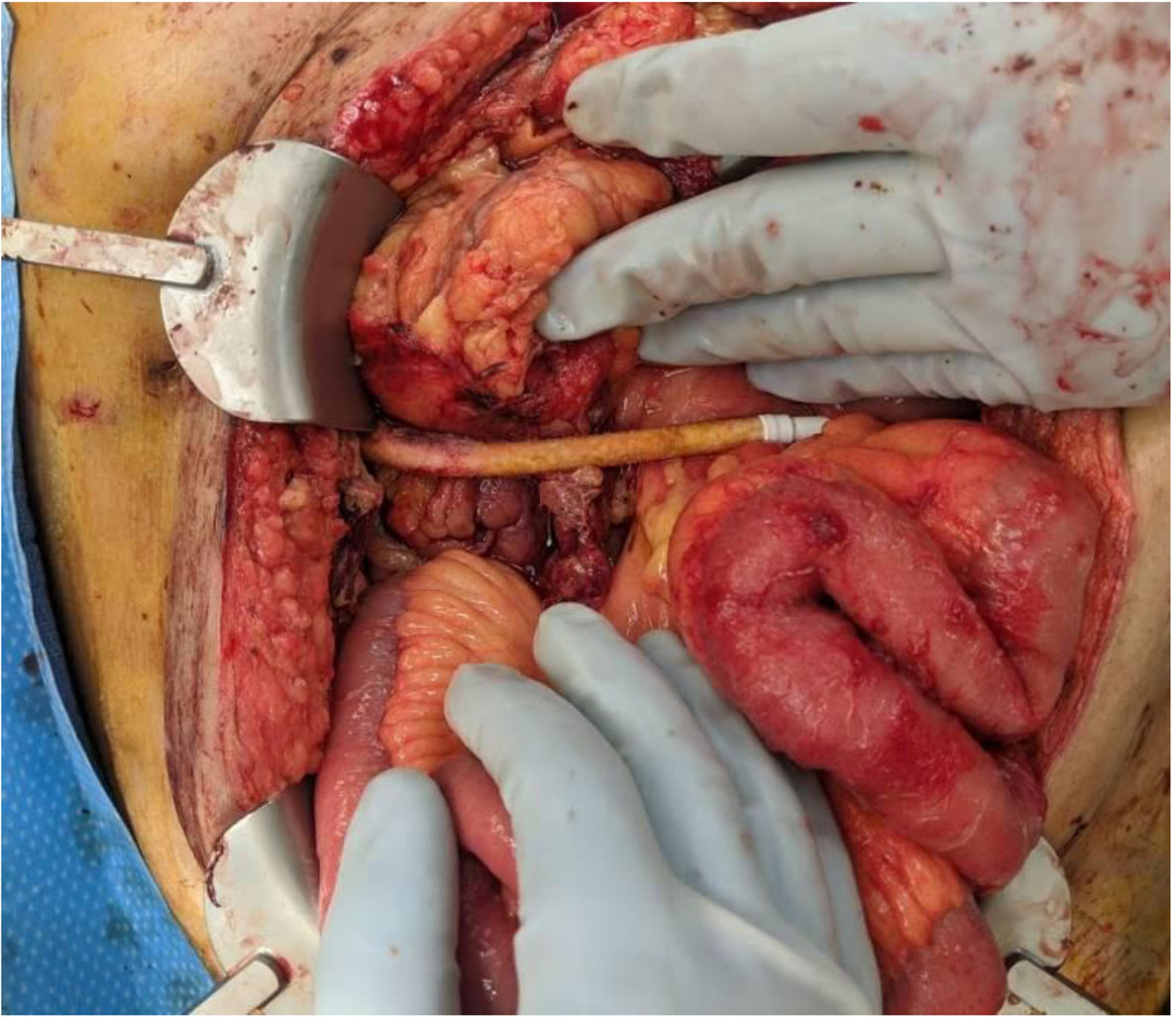
Intraperitoneal LVAD driveline traversing the abdomen

**Fig. 3:**
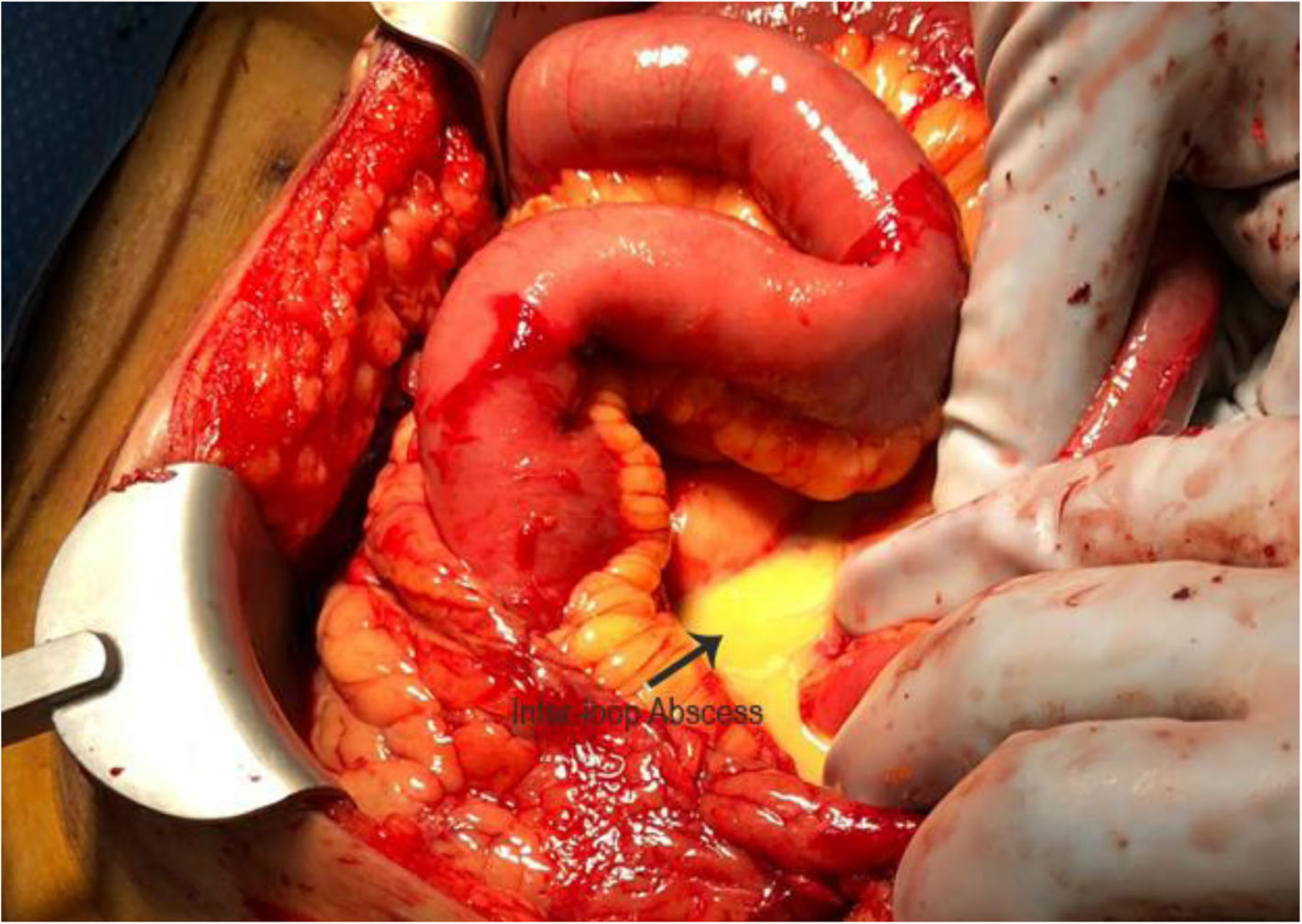
Purulent peritonitis with multiple inter-loop abscesses

Two days prior to removal of ECMO cannulas, the patient underwent right lower extremity guillotine amputation for dry gangrene due to iliac dissection and distal embolization. Five days later, on post LVAD day 25 there was noted to be stool leaking from the exit site of the driveline. The patient was taken to the operating room by the Cardiothoracic Surgery team and had a limited exploration, finding a bowel injury from the driveline. The injury was repaired with interrupted silk suture and the driveline was left untouched. Three days after this repair, Acute Care Surgery was consulted for stool leaking again from the driveline exit site (Fig. 1). The patient was found to be profoundly septic. He was started on antibiotics and taken to the operating room for formal exploratory laparotomy and was found to have the LVAD driveline traversing the abdominal cavity from the right upper quadrant to the left mid flank (Fig. 2). The driveline had injured the distal transverse colon and the prior repair was leaking. There was copious feculent fluid and numerous intra-abdominal abscesses that were drained (Fig. 3). Due to the patient’s sepsis, the colon was stapled proximal and distal to the injured segment and the specimen was removed. The patient was left in discontinuity and a temporary abdominal dressing was placed for a planned second look surgery. He returned to the ICU where he was given antibiotics, mechanical ventilation and vasopressors.

**Fig. 4:**
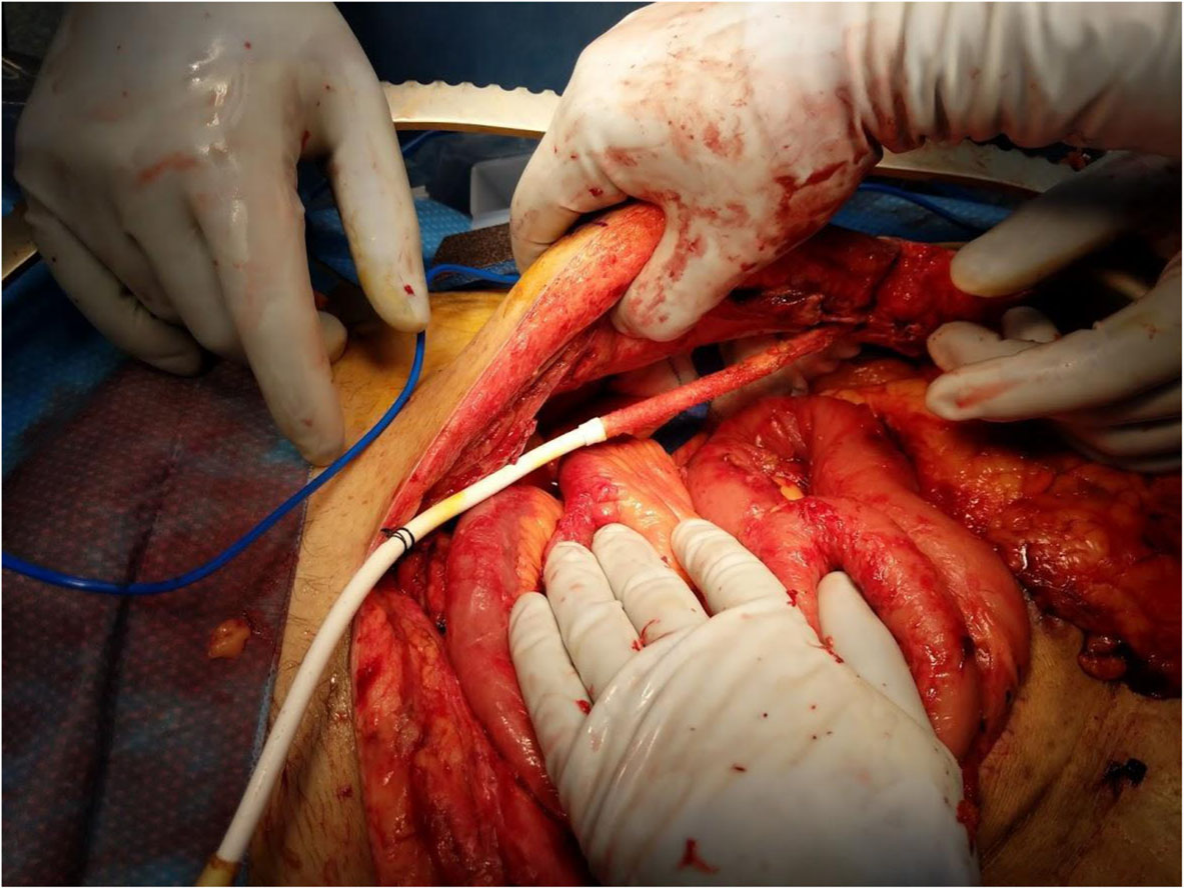
LVAD driveline brought from the left lower quadrant to the midline after opening the prior transverse abdominal incision on the patient’s left side

**Fig. 5:**
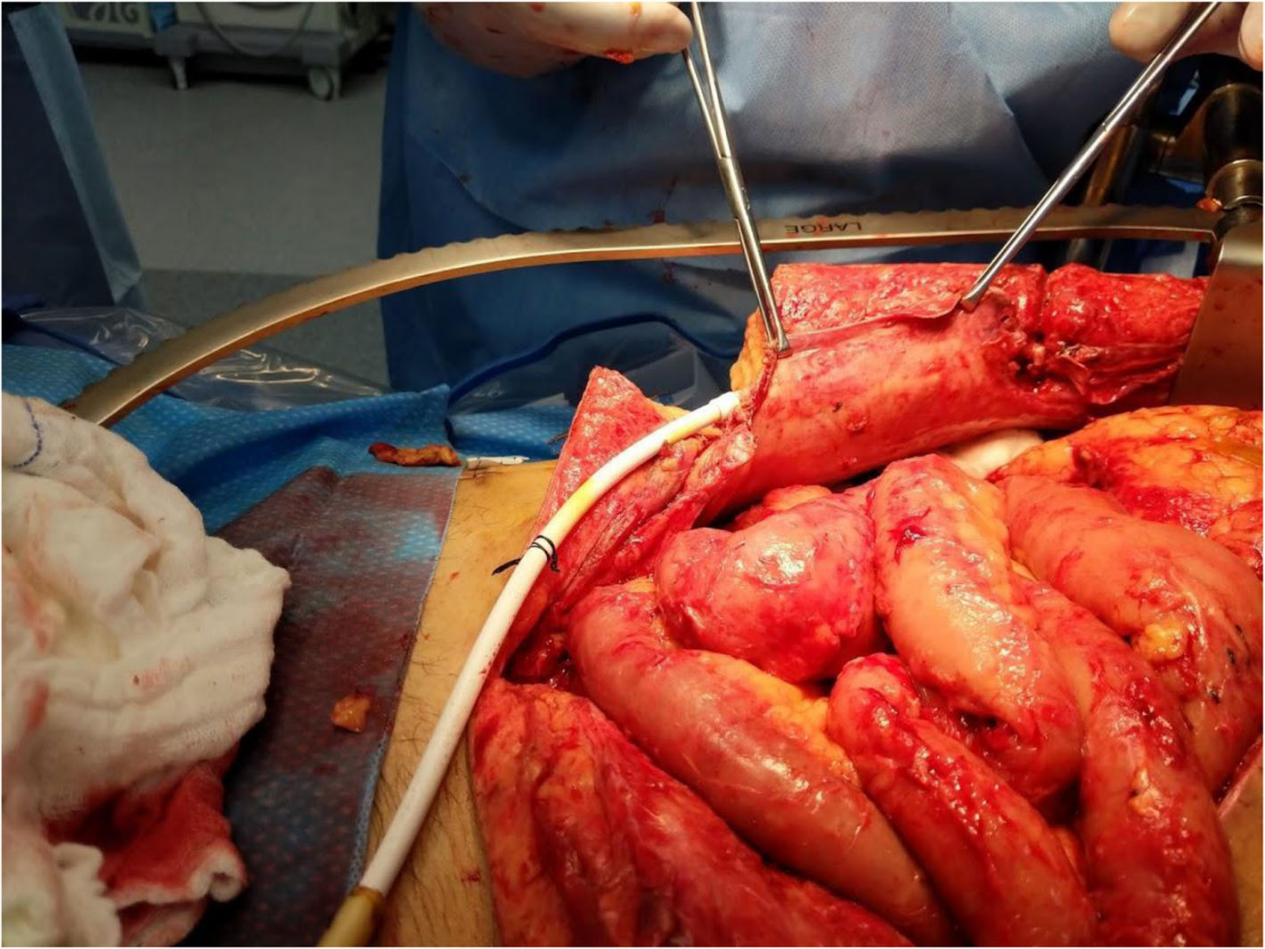
Retrorectus plane is developed and the driveline is extra-peritonealized

**Fig. 6:**
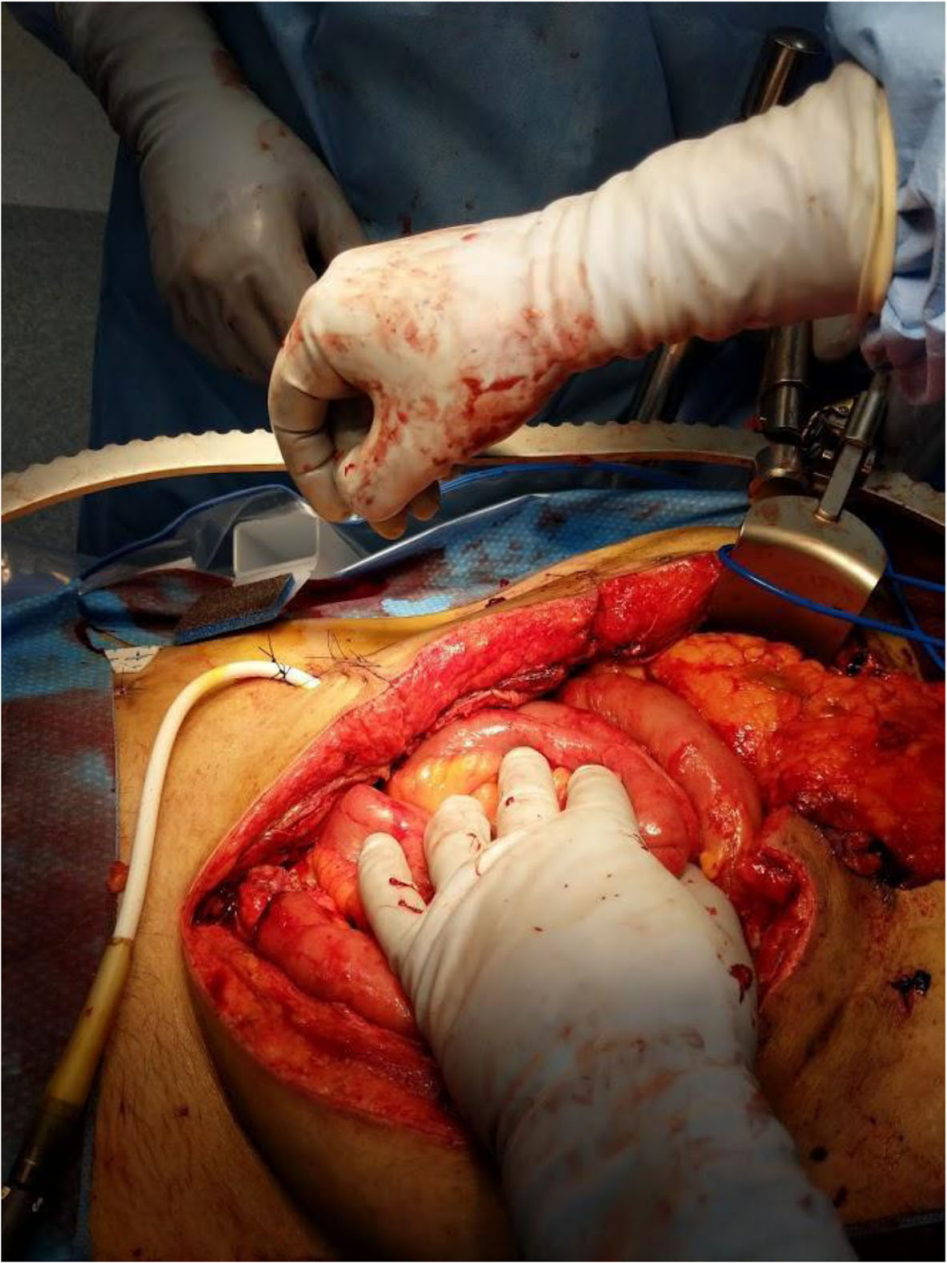
Final LVAD driveline position

**Fig. 7:**
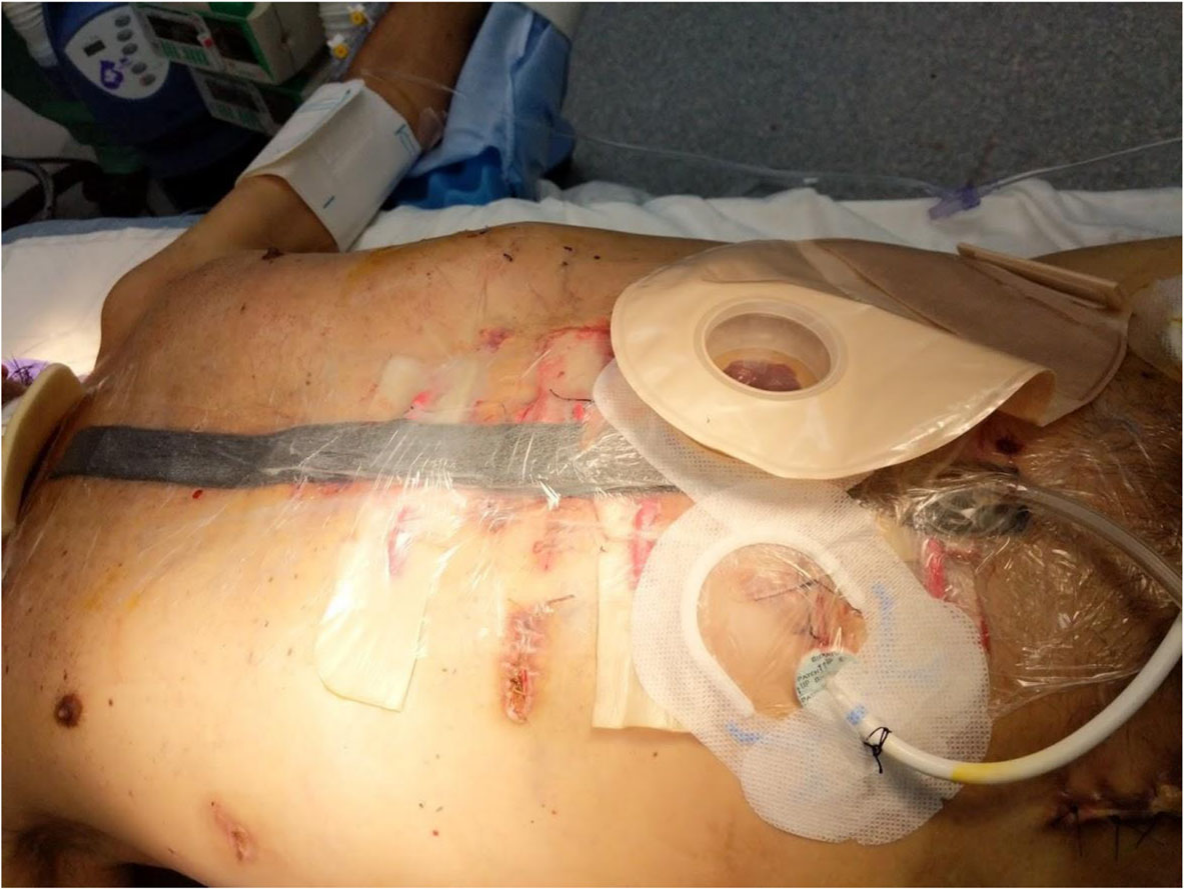
Post operative photo demonstrating LVAD exit site, ileostomy, retention sutures and incisional wound negative pressure dressing

Three days later, during the planned second look surgery the abdomen appeared clean. Due to the necessity of the LVAD, the driveline could not be disconnected. The first priority was to mobilize the driveline out of the peritoneum. The left side of the patient’s abdomen was opened transversely to allow the driveline to come to the middle (Fig. 4). The peritoneum was taken down from the right side of the patient’s abdomen to develop a retrorectus plane (Fig. 5) in an attempt to reposition the driveline to the intended extraperitoneal location. A smaller transverse incision was made on the right side to get the driveline in this retrorectus plane away from the midline (Fig. 6). Thus the driveline was successfully removed from the peritoneum without disconnection. A right hemicolectomy was performed, but due to the driveline exiting near the right lower quadrant, an end ileostomy was brought out in the left lower quadrant. His fascia was closed and retention sutures were placed due to the patient’s poor nutritional status and degree of abdominal sepsis (Fig. 7). The patient did well post operatively and was able to resume enteral nutrition on postoperative day 3, meeting his caloric demands by postoperative day 7. He underwent revision of his guillotine amputation 15 days after his last abdominal operation.

The patient’s course was complicated by an intra-abdominal abscess requiring interventional radiology drainage 3 weeks after his last abdominal surgery as well as prolonged IV antibiotic treatment for resistant organisms including VRE. The patient completed his antibiotic course and had his drains removed. At the time of this report, the patient remains hospitalized, tolerating enteral nutrition, off mechanical ventilation and undergoing physical therapy.

## Discussion and conclusion

As the population ages and the number of patients with heart failure increases, LVAD therapy is becoming increasingly common. The more known complications include bleeding, infection, pump thrombosis, right heart failure, device malfunction and stroke. Rarer, but devastating complications include bowel injury, bowel obstruction, fistula formation and hernia occurrence. To prevent driveline infection, the driveline is tunneled through the subcutaneous space and exits the patient’s abdomen to be connected to the controller. Inadvertent entry into the peritoneum can cause extreme morbidity and mortality to the patient. It is critical that the surgeon placing the LVAD takes the necessary precautions to prevent this [5].

This case demonstrates the importance of early recognition and involvement of an Acute Care Surgeon in the management of this complex problem. Appropriate treatment is paramount and involves a complete exploration, source control, driveline relocation and possible fecal diversion [6]. Although the incidence of this complication is low, it must be considered in the differential in a septic LVAD patient.

## Data Availability

Not applicable.

## Abbreviations

LVAD: Left ventricular Assist device
eCPR: Extracorporeal cardiopulmonary resuscitation
ECMO: Extracorporeal membrane oxygenation
AICD: Automatic implantable cardioverter-defibrillator
